# Morphology and articular configuration of the ceratopsid (Dinosauria: Ornithischia) lower hindlimb as revealed by a specimen from the Upper Cretaceous Dinosaur Park Formation of southern Alberta, Canada

**DOI:** 10.1371/journal.pone.0353362

**Published:** 2026-07-29

**Authors:** Brandon Theurer, Darren H. Tanke, Dylan Bastiaans, Khoi Nguyen, Philip Currie, Corwin Sullivan

**Affiliations:** 1 University of Alberta, Edmonton, Alberta, Canada; 2 Royal Tyrrell Museum of Palaeontology, Drumheller, Alberta, Canada; 3 Maastricht University, Maastricht, The Netherlands; 4 Natuurhistorisch Museum Maastricht, Maastricht, The Netherlands; 5 Philip J. Currie Dinosaur Museum, Wembley, Alberta, Canada; Soprintendenza Archeologia Belle Arti e Paesaggio Firenze Pistoia Prato, ITALY

## Abstract

Ceratopsid dinosaurs, such as *Triceratops*, are well-known for the striking horns and frills that are ubiquitous among members of the clade. But their postcranium has received little attention by comparison. Many questions persist regarding surprisingly fundamental aspects of the anatomy of the distal part of the hindlimb, such as the proper configuration of the metatarsals and the number and position of the distal tarsals. Here we use a taxonomically indeterminate but nearly complete lower hindlimb of a ceratopsid from the Upper Cretaceous Dinosaur Park Formation of southern Alberta, Canada, originally collected more than a century ago but never previously described, to address some of these uncertainties. The bones were CT scanned and segmented to create digital models that were then positioned based on morphological fit and comparisons with articulated specimens to establish the most plausible arrangement of the lower hindlimb elements. Proximally, the fibula articulated with the posterior surface of the laterally directed cnemial crest of the tibia and the lateral surface of the lateral condyle. The only distal tarsals present, distal tarsals III and IV, articulated with the proximal ends of the corresponding metatarsals, and their proximal surfaces were likely convex and concave, respectively. The metatarsals were closely appressed to one another throughout their lengths. The phalanges are asymmetric in varying ways and to varying degrees. Unguals of some ceratopsids have a visible shelf between the shaft and blade on the posterior side, while others, including the specimen described here, do not. The anatomical and arthrological insights obtained in this investigation of the distal part of the ceratopsid hindlimb will inform future biomechanical studies and contribute to comparisons of crural and pedal osteology among various ceratopsid taxa.

## Introduction

Ceratopsids are a clade of herbivorous dinosaurs known from the Late Cretaceous of North America that includes such iconic taxa as *Triceratops* and *Pachyrhinosaurus* [1]. Ceratopsids are notable for displaying extensive variation in such cranial features as horns, bosses, and frills, which provides most of the basis for their taxonomy [2]. The postcranium (skeletal elements except the skull and lower jaw) has accordingly received little attention in ceratopsids [3] despite the potential of postcranial elements to display some level of taxonomic variation and shed light on aspects of ceratopsid paleobiology, such as biomechanical performance and patterns of skeletal growth.

The relatively few studies to have considered ceratopsid postcranial elements (e.g., [4–11]) have left many details of the skeletal configuration unresolved, including a number pertaining to the distal part of the hindlimb. For example, many figures of naturally articulated specimens show the metatarsals closely appressed along their length (e.g., [5,12]), a condition sometimes described (e.g., [4]). Yet, many reconstructions of ceratopsid feet show the metatarsals splayed (e.g., [5,13,14]). There is also a need to clarify the arrangement of the distal tarsals in the articulated pes. Previous descriptions have provided varying, not always internally consistent accounts of tarsal placement [6] and of which distal tarsal is the largest (e.g., [5,12,13]). Additionally, even though the usual number of distal tarsals is two, there are occasional reports of three distal tarsals in some ceratopsid specimens [1,13].

A proper understanding of the articulation of all hindlimb elements, including the distal tarsals, is important not only for reconstructing the anatomy of the hindlimb but also for analyzing the ceratopsid kinematics during locomotion. The mobility of the dinosaurian ankle joint was typically concentrated at the contact between the proximal tarsals (astragalus and calcaneum) proximally and the distal tarsals and metatarsals distally [15], making the form and positioning of the distal tarsals important in determining the range of ankle motion.

Here we describe the distal part of the ceratopsid hindlimb in detail based on a taxonomically indeterminate but nearly complete ceratopsid crus and pes from the Upper Cretaceous Dinosaur Park Formation of Dinosaur Provincial Park in southern Alberta, Canada, interpreted in the light of comparisons to other specimens, including skeletons preserved in natural articulation. We build on previous work by providing the first detailed description of the osteology and inferred articular configuration of a ceratopsid lower hindlimb.

Institutional Abbreviations: AMNH FARB (American Museum of Natural History Fossil Amphibians, Reptiles, and Birds, New York, USA); CMN (Canadian Museum of Nature, Ottawa, Canada); MOR (Museum of the Rockies, Bozeman, USA), ROM (Royal Ontario Museum, Toronto, Canada); TMP (Royal Tyrrell Museum of Palaeontology, Drumheller, Canada); UALVP (University of Alberta Laboratory for Vertebrate Palaeontology, Edmonton, Canada); UMNH (Natural History Museum of Utah, Salt Lake City, USA); USNM (United States National Museum of Natural History, Washington DC, USA).

## Materials and methods

**Fig 1:**
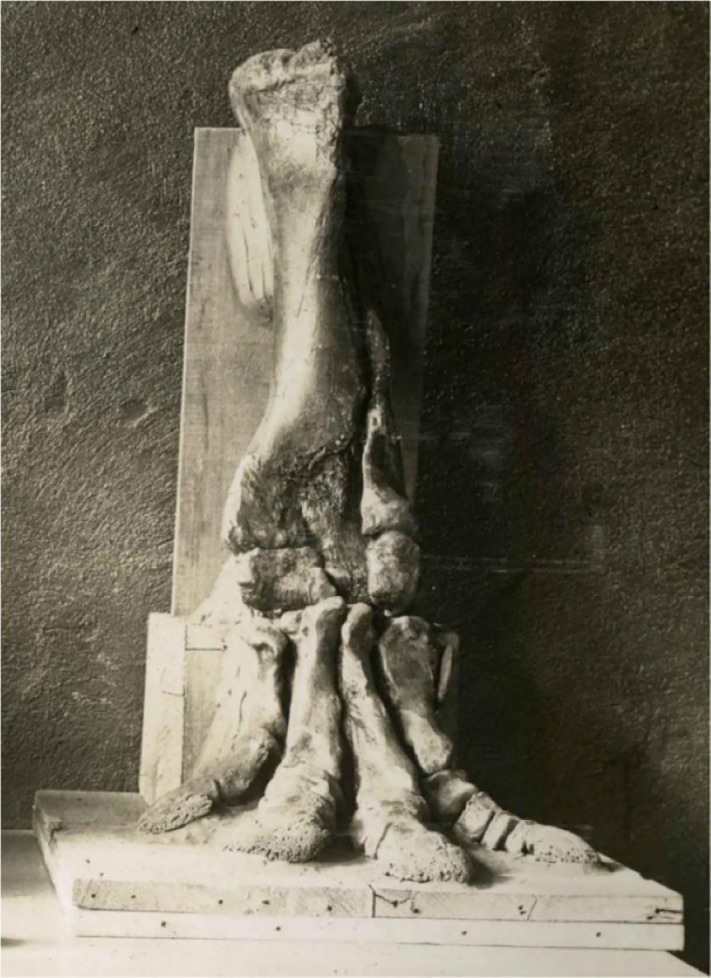
Mounted lower hindlimb specimen. UALVP 42, the left lower hindlimb of an indeterminate ceratopsid dinosaur, as mounted by Sternberg in the early 1920s.

The specimen described in this paper, UALVP 42 (Fig 1), was excavated by private collector George F. Sternberg in 1920. Sternberg subsequently sold his collection to the University of Alberta in Edmonton, Canada. UALVP 42 is a left crus and pes comprising all elements distal to the knee joint other than phalanx I-1, although some bones are incomplete [16]. Sternberg’s field notes state that a large portion of the skeleton was too weathered to collect.

**Fig 2:**
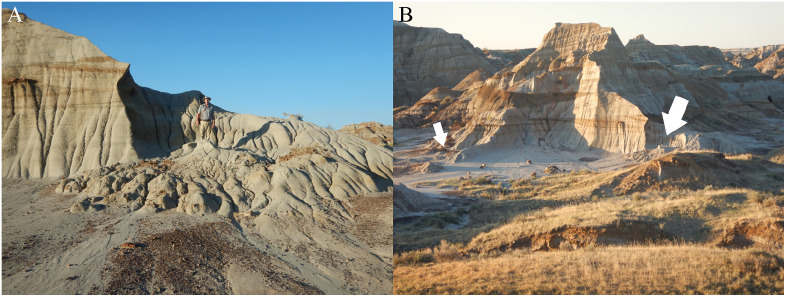
Ceratopsid mystery quarry [UTM 12U 464163; 5621193 (WGS 84)] in sandstone. (A) Close up photo of the quarry interpreted as having yielded UALVP 42. One of the authors (DHT) stands behind remnants of a plaster jacket pedestal. Dinosaur Provincial Park, looking roughly east, May 15, 2023. Despite 103 years of erosion, the quarry and waste rock scree slope are still readily visible. Photo by Mark T. Mitchell. (B) The ceratopsid mystery quarry (large arrow) interpreted as having yielded UALVP 42, and 1913-1914 American Museum of Natural History ankylosaur quarry (AMNH FARB 5337). The ankylosaur quarry was reopened by TMP in 2018 (TMP 2018.012.0151; indicated by the small arrow). Dinosaur Provincial Park, looking roughly north, May 15, 2023. Photo by Darren H. Tanke.

The specimen was presumably prepared and completely extracted from the rock by Sternberg; it appears fully prepared and mounted in an April 1, 1922 Edmonton newspaper article [17]. As with many other significant fossils collected during the Great Canadian Dinosaur Rush in Alberta during the first half of the 20^th^ Century, precise locality information for this important specimen is unfortunately lacking. Absence of such data negatively impacts the scientific value of research specimens, especially in making it impossible to establish their exact stratigraphic provenance. The early dinosaur hunters often seemed more concerned with collecting fossils than with accurately recording where they came from, despite the availability of basic topographic maps which would have enabled them to do so. However, missing provenance data can sometimes be recovered by various sleuthing techniques. Ongoing successful lost quarry relocation efforts by one of us (DHT) and analysis of unidentified “mystery quarries” in Dinosaur Provincial Park (i.e., [18–21]) and various other lines of evidence have narrowed down the search area for the UALVP 42 discovery site. Several previously undocumented dinosaur quarries worked by Sternberg in 1920 within the central core of what is now Dinosaur Provincial Park have been successfully identified [22–24], and occur in a relatively localized field area. Their occurrence in a tight cluster reflects the logistical advantages of working multiple quarries in a restricted area in any given year. In the badlands worked by Sternberg in 1920 is a mystery quarry with badly eroded *ex situ* ceratopsid bones remaining on the quarry floor and more in the excavation scree on the slope below the quarry. When the quarry was rediscovered in 1980, a single, eroded rock pedestal (Fig 2A) indicated a plaster jacket containing bones had been collected there. In 1993, an *in situ* ceratopsid posterior dorsal rib (TMP 1993.036.0076) was recovered by one of us (DHT) at the site.

The site is just 165 m north of a relocated and confirmed (based on field photographs taken during the original excavation) 1920 George Sternberg quarry (*Chasmosaurus* UALVP 40, quarry 237). Sternberg’s field notes record that UALVP 42 was found “close” to UALVP 40, but provide no direction or distance measurements. The American Museum of Natural History extensively worked this same area in 1913–1914, and one of their quarries is just 31 m from the ceratopsid mystery quarry [25] (Fig 2B). Despite thorough research, there is no evidence that the ceratopsid mystery quarry was ever worked by them, though they must have seen it; all of their major ceratopsid specimens were collected elsewhere [19,22,26]. Locations where articulated ceratopsid skeletons, or parts thereof, were collected by other institutions likewise do not match the mystery quarry [27]. No known ceratopsid quarries other than the mystery quarry occur “close” to the UALVP 40 site. Major ceratopsid quarries with articulated postcrania are relatively uncommon in Dinosaur Provincial Park. Most are documented with a data-bearing metal quarry stake set in concrete at the site [28] and/or survey-grade GPS mapping [29], providing accurate locality data, and none match the mystery quarry. Another clue is the quality of bone preservation. Dinosaur limb bones preserved in clay are often crushed to varying degrees as a result of dewatering and compaction of the wet clay by heavy overlying sediments. The condition of the bones of UALVP 42 suggests the specimen was found in sandstone; Sternberg’s field notes say, “The rock is fine,” and the mystery quarry is in good and firm sandstone rock. Finally, the fact that UALVP 40 and UALVP 42 are closely similar catalogue numbers suggests the two specimens may have been found relatively close together, although there is less certainty on this point.

With the above points in mind, by process of elimination and in the absence of any evidence to the contrary, we posit the mystery ceratopsid quarry as the site that yielded UALVP 42. If so, the remnants of bone on the quarry floor and downslope show Sternberg uncovered a partial and partly scattered skeleton, and because of its poor and weathered condition, collected the lower hindlimb only. The tendency of early collectors to take only selected skeletal parts, whether they were “head hunting” or engaging in the less common “limb hunting” seen in this particular case, adds to the confusion regarding where, when, and by whom certain historical specimens were collected [18,24,30,31], although the provenance of UALVP 42 now seems secure. The quarry is about 8 m above the Oldman/Dinosaur Park Formation contact, putting it firmly in the *Centrosaurus*-*Corythosaurus* ceratopsid/hadrosaurid faunal zone and thus making UALVP 42 likely referable to either *Centrosaurus* or *Chasmosaurus* [32].

For display purposes, the missing portions of the bones were reconstructed, presumably by Sternberg, using plaster mixed with brown paint, a material difficult to distinguish from the original bone. Furthermore, Sternberg mounted the bones with a ceratopsid metacarpal in place of the missing phalanx I-1. It is possible that the metacarpal belongs to UALVP 42, as Sternberg’s field notes indicate one “phalange” was out of place and likely originated from digit I or II. It is possible that this displaced “phalange” is the metacarpal substituted for phalanx I-1 in the mount. However, the metacarpal differs slightly in color from the bones that undoubtedly belong to UALVP 42, which may indicate it was preserved under different conditions and may be from a different specimen preserved at another location. Regardless, comparison with *Styracosaurus* CMN 344, as shown in [7], reveals strong similarity to metacarpal IV in that specimen, as noted by [16]*.* The metacarpal will not be considered further in this paper. At some point, the mount of UALVP 42 was disassembled, except that the tibia and fibula remain held together by a metal bar, the fibula and calcaneum are closely appressed and possibly fused, and metatarsal IV and V are held together by a metal bar.

To distinguish unambiguously between reconstructed and original parts of the specimen for our reconstruction of the distal hindlimb, and to create renderings of the bones that could be digitally manipulated, UALVP 42 was CT-scanned using a Siemens Somatom Definition Flash scanner at the University of Alberta Hospital and segmented using Dragonfly ORS [16]. The scans are available on Figshare (https://doi.org/10.6084/m9.figshare.32745912). Because bone is much more radiodense than plaster, it shows up brighter in CT images. An initial segmentation of the original fossil bone was performed using a point-and-click feature based on a defined range of radiodensities. This initial segmentation was then manually refined, slice by slice, to remove any plaster that had accidentally been included due to an artifactual increase in its radiodensity (for example, due to edge hardening, a phenomenon which causes the edges of an object to appear more radiodense than the rest of the object).

**Fig 3:**
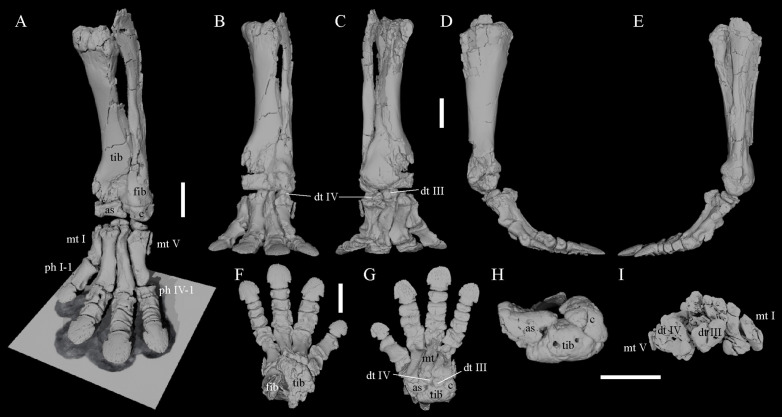
Articulated elements of UALVP 42 (lower hindlimb from an indeterminate ceratopsid), shown as preserved without restoration or retrodeformation of the individual bones. All scale bars = 10 cm. Hindlimb shown in (A) oblique anterolateral view with footprint modified from Fig 10F in [33]. Panels B-G show orthographic (B) anterior, (C) posterior, (D) medial, (E) lateral, (F) proximal, and (G) distal views. Panels H-I show perspective views. (H) Distal view of the articulated tibia, astragalus, and calcaneum showing the proximal articular surface of the ankle joint (updated version of Fig 7D in [16]). (I) Proximal view of the articulated distal tarsals and metatarsals, showing the distal articular surface of the ankle joint. Abbreviations: as = astragalus. c = calcaneum. fib = fibula. mt I = metatarsal I. mt V = metatarsal V. ph I-1 = phalanx 1-I. ph IV-1 = phalanx IV-1. tib = tibia.

Parts of both ends of the tibia proved heavily reconstructed, but other elements, such as the tarsals, are almost entirely genuine. The segmented renderings of the bones in their unreconstructed state were imported into Autodesk Maya to create a 3D model of the articulated distal part of the hindlimb (Fig 3) [16]. The most plausible articular configuration for the model was determined based on comparison with specimens preserving the natural articulation of the lower hindlimb elements, such as an indeterminate chasmosaurine CMN 8547 and *Centrosaurus* AMNH FARB 5351 (note that according to [5] the articulation of the left hindlimb and foot of AMNH FARB 5351 was based on another specimen that preserved the articulation of these elements), the apparent goodness of fit of the bones (avoiding unrealistic gaps between elements), and the necessity of preventing bones from overlapping and thus creating an impossible geometry. The iterative process involved in this was described in [16]. As noted previously, the proximal phalanx of digit I is missing from the foot of UALVP 42. Thus, the left phalanx I-1 from another specimen (UALVP 16248, a partially articulated *Centrosaurus* skeleton from the Dinosaur Park Formation) was used in the reconstruction, as described previously [16].

**Fig 4:**
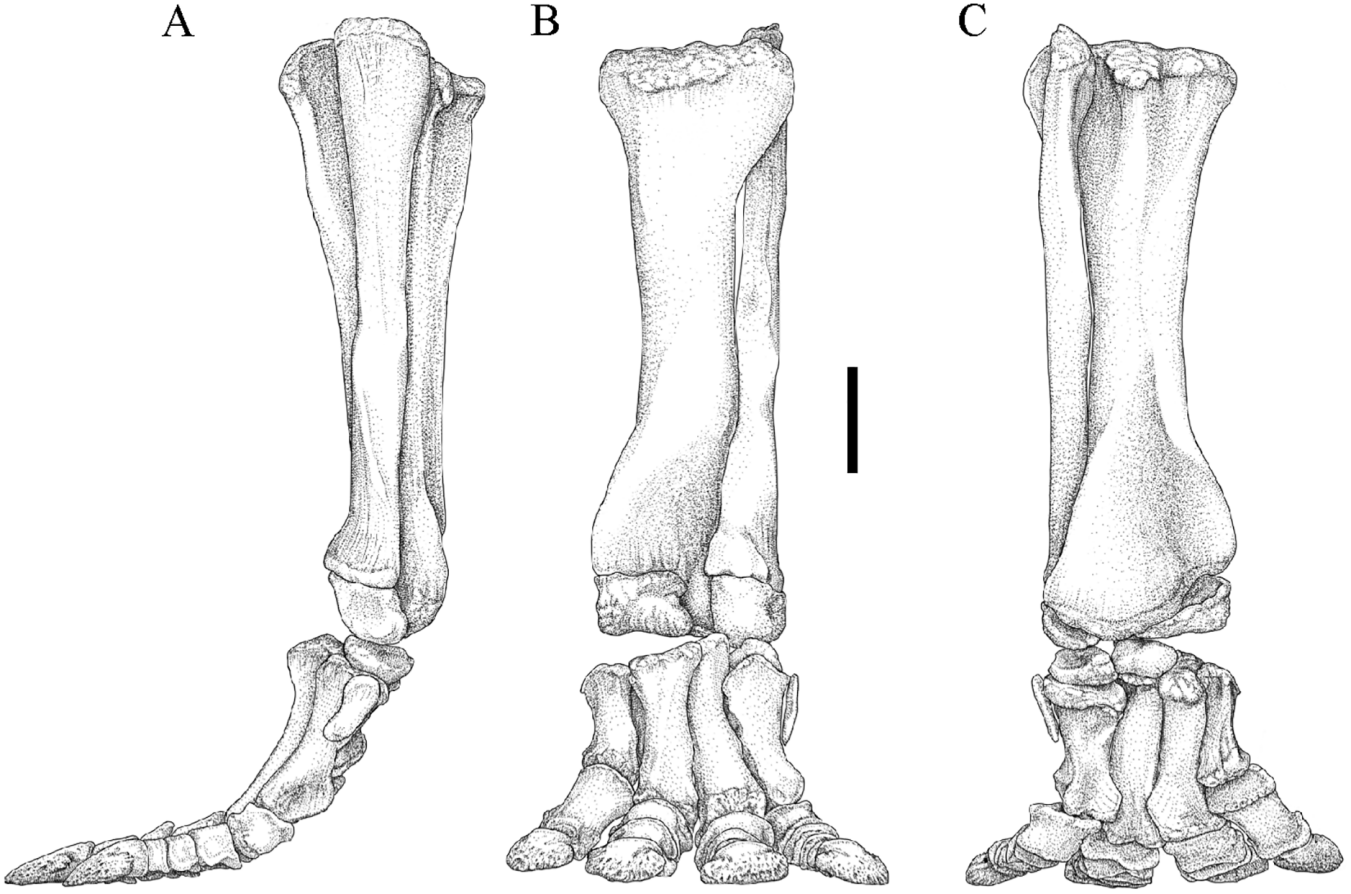
Orthographic illustration reconstructing the articulated elements of UALVP 42. Shown in (A) lateral, (B) anterior, and (C) posterior views.

We provide an illustration of UALVP 42 (Fig 4) as reconstructed based on both the 3D model incorporating UALVP 16248 and a comparison with another partially articulated *Centrosaurus* (UALVP 55261) from the Belly River group of White Rock Coulee in southeastern Alberta. UALVP 55261 has multiple overlapping elements with UALVP 42, such as the tibia, fibula, and metatarsals I-III. While the tibia and fibula are slightly crushed mediolaterally, the specimen is still better preserved than UALVP 42, particularly at the proximal and distal ends of the tibia. Even though some aspects of tibial morphology generally differ between chasmosaurines and centrosaurines as discussed below, using *Centrosaurus* to supplement our understanding of UALVP 42 seems reasonable because UALVP 42 is taxonomically indeterminate and the differences are usually subtle.

Directional terms used in the descriptions assume that the animal is holding the entire hindlimb, including the toes, straight out below its body so that the distal aspect of each bone points ventrally. In figures showing proximal or distal views, the anterior side of each bone is directed upward. Measurements were taken of the maximal extent of the bone in the dimension specified (e.g., proximalmost point to distalmost point for a bone length) using the distance tool in Maya. No permits were required for the described study, which complied with all relevant regulations.

## Results

### Osteology

#### Tibia.

**Fig 5:**
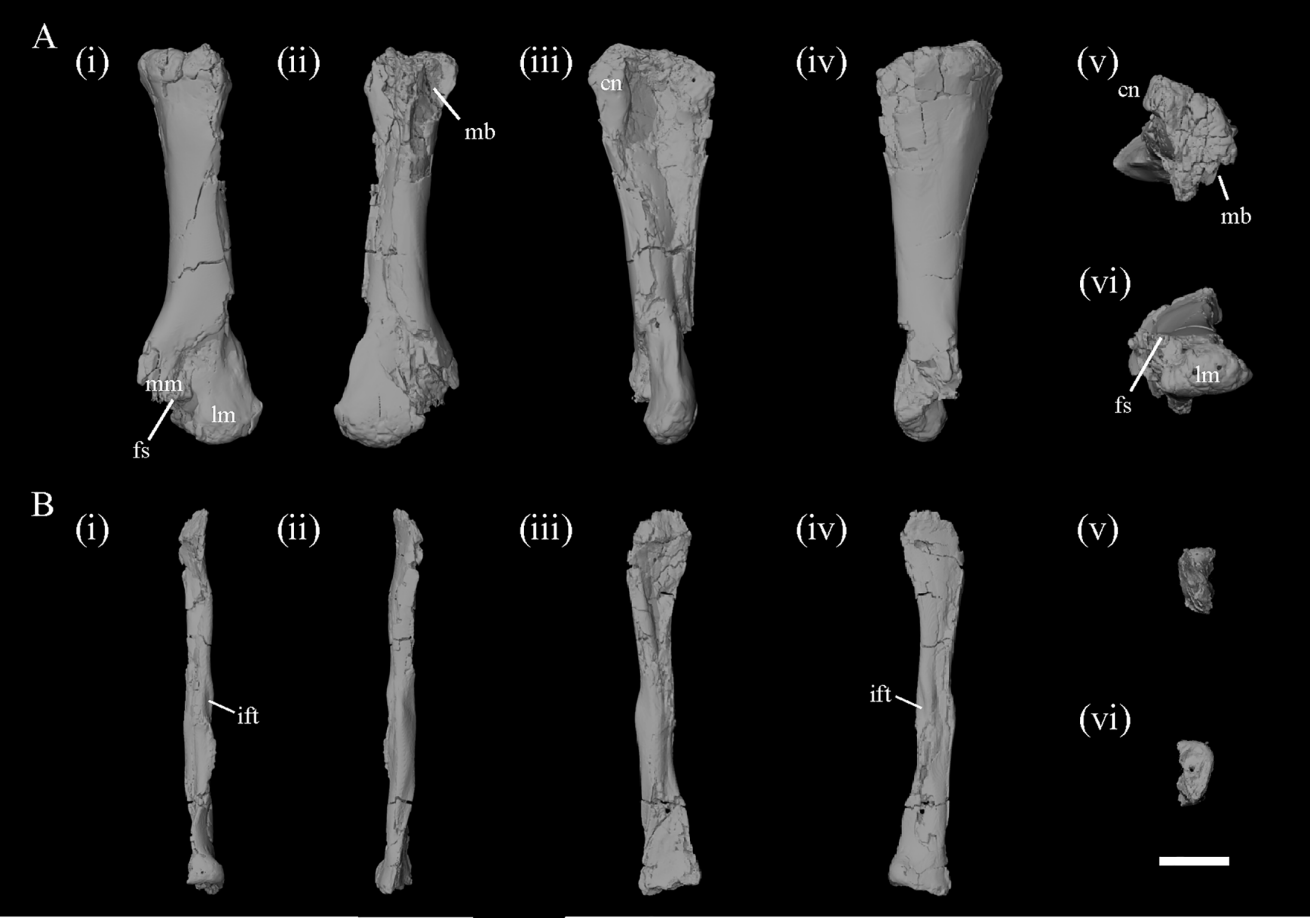
Orthographic views of the crural elements of the indeterminate ceratopsid UALVP 42. Scale bar = 10 cm. (A) Tibia. (B) Fibula. Each shown in (i) anterior, (ii) posterior, (iii) medial, (iv) lateral, (v) proximal, and (vi) distal views. Abbreviations: cn = cnemial crest. fs = flat surface of anterior part of distal end of medial malleolus. ift = homolog of iliofibularis tubercle. lm = lateral malleolus. mb = area of missing bone. mm = medial malleolus.

The tibia (Fig 5A) in UALVP 42 is an elongate bone measuring 56.9 cm in length, with expanded proximal and distal ends (Table 1). The proximal 2/3 of the lateral side of the shaft of the tibia has been crushed in postmortem. The crushing continues diagonally around the posterior side of the bone to the vicinity of the damaged medial malleolus. The medial side of the shaft is smoothly rounded with apparently no natural ridges.

**Table 1:** Measurements of crural bones of UALVP 42.

Element	Length (Proximodistal) (mm)	Proximal End (mm)		Midpoint (mm)		Distal End (mm)	
**Width (Medio-lateral)**	**Depth (Antero-posterior)**	**Width (Medio-lateral)**	**Depth (Antero-posterior)**	**Width (Medio-lateral)**	**Depth (Antero-posterior)**
Tibia	568.6	89.6	184.5	70.3	112.2	–	71.6
Fibula	545.6	25.3	87.6	36.5	54.6	51.2	93.7

Note: The distal end of the tibia is too damaged to measure an accurate mediolateral width.

The proximal end is expanded anteroposteriorly while the distal end is expanded mediolaterally, but this difference seems to be due to postmortem deformation. The proximal ends of most ceratopsid tibiae are expanded mediolaterally like their distal ends, with only a slight angular offset between the directions of expansion of the two ends (e.g., *Centrosaurus* UALVP 55261 and *Pachyrhinosaurus* UALVP 57285). The atypical morphology of UALVP 42 is unlikely to reflect taxonomic variation, as some species, like *Pachyrhinosaurus lakustai*, offer examples of usual (e.g., UALVP 57285), deformed (e.g., UALVP 61545), and even intermediate conditions (e.g., TMP 1989.055.1093).

The proximal end of a typical ceratopsid tibia has three prominences: the medial and lateral condyles for articulation with the femur, and a lateral flange that is likely the cnemial crest (e.g., [4,8,10], but see [6,9] for other interpretations). However, these prominences have been severely damaged or lost in UALVP 42 due to the deformation of the proximal end of the tibia. The cnemial crest has been crushed mediolaterally but still protrudes slightly in the lateral direction, and the medial condyle is completely missing. The posteriormost preserved part of the proximal end may represent the lateral condyle. However, deformation of the proximal end makes this identification dubious. In undeformed specimens (e.g., *Centrosaurus* UALVP 55261, *Chasmosaurus* UALVP 52613), the articular condyles are rounded projections that are subequal in size, and the lateral flange is a comparatively thin blade that projects laterally from the proximal end of the bone.

As in most ornithischians (e.g., [34–43]), including other ceratopsids (e.g., *Centrosaurus* UALVP 55261, *Chasmosaurus* UALVP 52613), the distal end of the tibia is expanded transversely, and the lateral side of the distal end extends farther distally than the medial side, creating a rounded and protruding lateral malleolus. The lateral malleolus of UALVP 42 is separated from the weathered and incomplete medial malleolus by a deep groove on the anterior surface of the tibia. The anterior part of the medial malleolus forms a flat, distally facing surface for articulation with the astragalus. The anterior surface of the distal part of the tibia is gently concave, although this may be exaggerated as a result of crushing. The posterior surface is strongly convex, but this curvature is preserved only laterally.

#### Fibula.

The proximal end and much of the shaft of the fibula (Fig 5B) are mediolaterally compressed as in other ceratopsids, although this seems to be exaggerated near the proximal end by a broad artifactual depression on the medial surface. The proximal surface of the fibula is tilted slightly medially and is convex anteroposteriorly. At approximately the midpoint of the shaft is an anteriorly directed facet that is shallowly concave, and may represent a homolog of the iliofibularis tubercle present in many other archosaurs [44]. Opposite this facet, on the posterior side of the fibula, is the proximal end of a broad, posteriorly directed surface that ends slightly proximal to the distal end of the bone. The proximal and distal portions of this surface are slightly concave, while the middle portion is flat, and the distal portion is bordered by a sharp lateral edge that ends abruptly and a sharp medial edge that continues to the end of the bone. The shaft of the bone twists slightly at the level of this distal concave area, such that the distal end of the fibula is slightly expanded along an axis extending from anteromedial to posterolateral. The expansion and the overall mediolateral compression of the bone contribute to a flat, longitudinally striated, posteromedially directed surface that would have rested against the tibia. The distal end of the fibular shaft also bears an anterior bulge, which makes the distal surface of the fibula teardrop-shaped with a narrow posterior end. The distal end seems to be concave, although in some places the quality of the CT data does not allow the fibula to be readily distinguished from the attached calcaneum. However, a concave distal end would match the condition in chasmosaurines (*Triceratops* ROM 1434, *Utahceratops* UMNH 12198, *Vagaceratops* CMN 41357). Centrosaurines (e.g., *Centrosaurus* ROM 1426; *Pachyrhinosaurus* TMP 1987.055.0192 and TMP 1989.055.0555) also have a concavity on the distal end of the fibula, but the concavity is restricted to at most the anterior half of the distal surface.

#### Astragalus.

**Fig 6:**
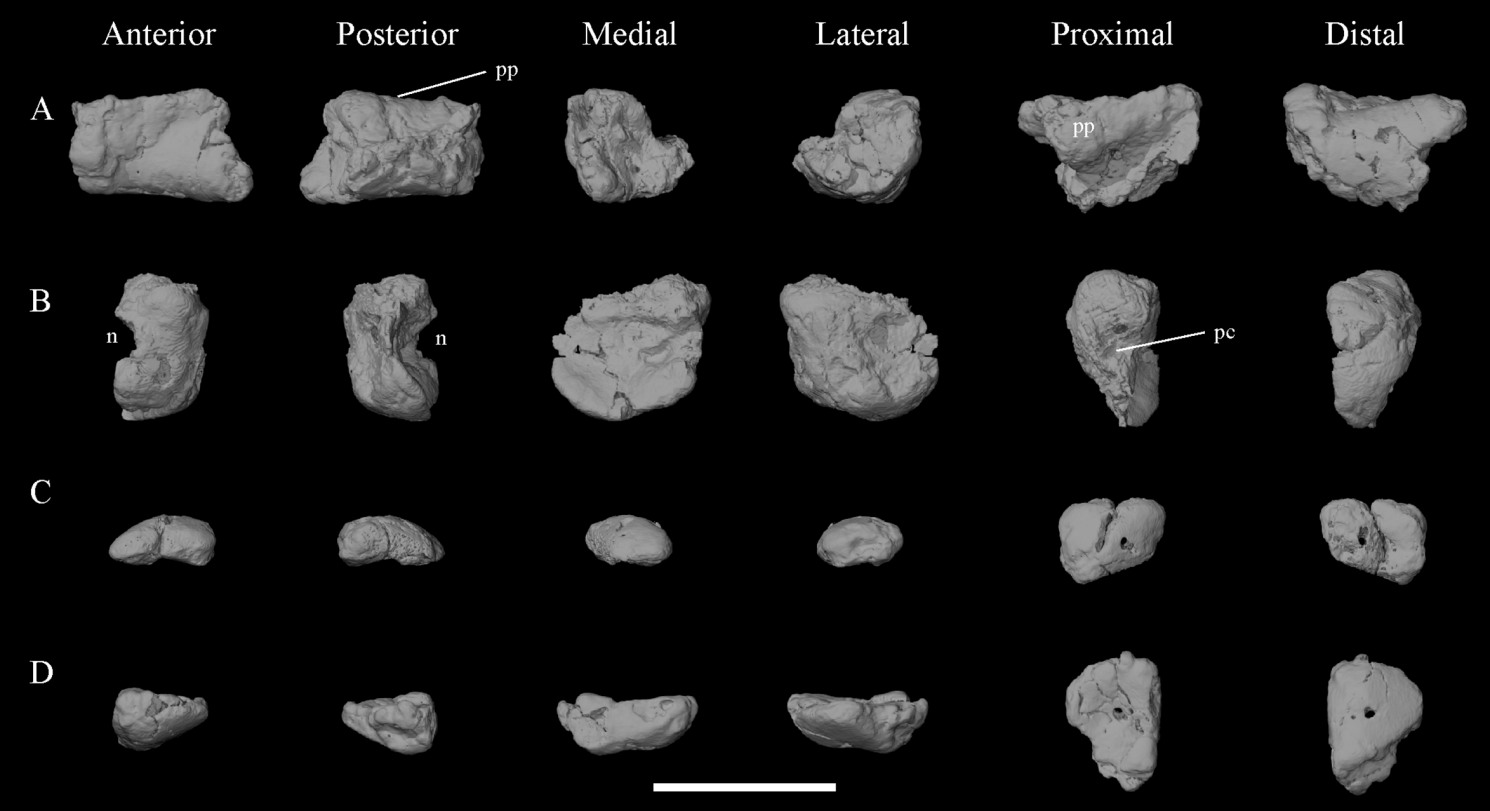
Orthographic views of the tarsals of the indeterminate ceratopsid UALVP 42. Scale bar = 10 cm. Shown in anterior, posterior, medial, lateral, proximal, and distal views. (A) Astragalus. (B) Calcaneum. (C) Distal tarsal III. (D) Distal tarsal IV. Abbreviations: n = notch. pc = concavity on the posterior part of the proximal surface. pp = proximally prominent portion of the proximal end.

The astragalus (Fig 6A) is an irregularly shaped bone that is mediolaterally wide and roughly equal in proximodistal height and anteroposterior thickness (Table 2). The lateral part of the posterior surface has been lost taphonomically. The anterior surface is broad and smooth, although slightly concave mediolaterally. The distal surface is convex anteroposteriorly, and this curvature continues onto the distal part of the posterior surface. The proximal face of the astragalus has two distinct portions, one more proximally prominent than the other. The more prominent portion is more anteriorly positioned and slightly wedge-shaped, broadening medially. The surface is essentially flat, slanting slightly laterally, and has a natural fit with the flat surface on the distal face of the medial malleolus of the tibia. The less proximally prominent portion of the astragalus is posteriorly positioned and strongly slanted medially.

**Table 2:** Measurements of the tarsal bones of UALVP 42.

Element	Height (Proximodistal) (mm)	Width (Mediolateral) (mm)	Depth (Anteroposterior) (mm)
Calcaneum	75.7	43.8	79.5
Astragalus	62.6	95.0	63.8
Distal Tarsal III	20.4	60.7	39.7
Distal Tarsal IV	33.4	51.6	77.4

Note: Height was measured in the middle of the bone (i.e., in the middle of the distal concavity) for distal tarsal III and near the medial side for distal tarsal IV.

The posterior surface of the astragalus is proximodistally short and has a sharply defined proximal edge that forms a relatively straight diagonal line extending from proximomedial to distolateral, though the middle portion of this edge is missing. The oblique orientation of the proximal edge is unusual because the corresponding feature in other ceratopsids seems to consistently either extend subhorizontally for some distance from the proximomedial corner of the posterior surface and then plunge obliquely towards the distolateral corner, forming an angle between the two line segments (*Centrosaurus* CMN 57053, *Chasmosaurus* ROM 843, indeterminate chasmosaurine CMN 8547), or follow a convex arc from the distomedial corner to the distolateral corner (e.g., *Chasmosaurus* UALVP 52613 and CMN 2245, indeterminate centrosaurine CMN 8896, *Vagaceratops* CMN 41357). In all ceratopsids that preserve the astragalus in articulation with the tibia, there is a close fit between the two bones, suggesting that the morphology of the astragalus could inform the proper restoration of the lateral malleolus of the tibia in UALVP 42.

A subtriangular projection protrudes laterally from the distal part of the anterolateral edge of the astragalus and helps to define a concavity on the lateral surface. Combined with the slight concavity of the anterior surface of the astragalus, this lateral projection gives the astragalus a somewhat crescentic outline in distal view. The medial surface of the astragalus is comparatively flat but bears a slight depression near the proximal edge.

#### Calcaneum.

The calcaneum (Fig 6B) has the overall shape of a concave disk. As with the fibula, the anterior part of the calcaneum is mediolaterally wider than the posterior part. The distal face is convex, like those of the astragalus and the lateral malleolus of the tibia. By contrast, the medial surface of the calcaneum is strongly concave, and the broad, subcircular lateral surface of the calcaneum is slightly concave. The anteromedial edge of the calcaneum bears a notch, which is clearly apparent in anterior view. The notch has a close counterpart in other specimens, such as UALVP 55261 (*Centrosaurus*), and therefore likely represents a consistent morphological feature rather than simply a taphonomic artifact or a product of individual variation. The notch is in fact quite helpful in distinguishing the calcaneum from the distal tarsals and from partial or distorted vertebrae. Interestingly, the calcaneum of USNM V 7957 (a largely complete juvenile centrosaurine left pes originally referred to “*Brachyceratops*”) seems to lack this distinctive notch, suggesting that it may have developed ontogenically as an individual matured.

The proximal surface of the calcaneum is concave posteriorly, but convex anteriorly. Over much of the contact between the calcaneum and fibula, the two bones are closely appressed but not fused. Near the center of the contact, however, it is difficult to identify a boundary between the two bones, perhaps because of incipient fusion. Various types and degrees of fusion have previously been reported for the crural and proximal tarsal elements of ceratopsians, including between the calcaneum and astragalus [6,45–47], astragalus and tibia [4,5,13,48–50], and calcaneum and tibia [9]. However, fusion between the calcaneum and the fibula has not previously been reported to our knowledge.

#### Distal tarsals.

UALVP 42 has two distal tarsals, almost certainly representing distal tarsals III (Fig 6C) and IV (Fig 6D). Although [13] described and illustrated a configuration with three distal tarsals in “*Brachyceratops*” USNM V 7956 and “*Brachyceratops*” USNM V 7957, with the largest distal tarsal contacting metatarsal II, the smallest contacting metatarsal III, and the third contacting metatarsal IV, reexamination of USNM V 7597 suggests that the largest of the three purported “distal tarsals” is the misidentified calcaneum. Additionally, [13] described a calcaneum, but did not illustrate this element except as a purported distal tarsal, and mentioned only two distal tarsals in the first sentence of his description of the tarsus (despite going on to describe three) and when listing the bones present in USNM V 7597, the paratype of “*Brachyceratops*”. Thus, it seems safe to conclude that confusion crept into the treatment of the tarsus of USNM V 7597 in [13], and that the general condition for ceratopsid dinosaurs is to have ossified third and fourth distal tarsals, as in typical archosaurs.

Although the orientations of the distal tarsals are somewhat uncertain, the following description is based on the configuration we consider most likely to be correct. Distal tarsal III is smaller than distal tarsal IV. Each distal tarsal has a strongly convex face opposite a concave face, but the convex face is directed distally in the larger distal tarsal and proximally in the smaller distal tarsal. Likewise, each distal tarsal has a proximodistally thin edge opposite a proximodistally thicker one, but the thicker edge is lateral in distal tarsal III and medial in distal tarsal IV.

Distal tarsal III (Fig 6C) is sub-rectangular in proximal view. The midregion of its proximodistally thickened lateral edge bears a small concavity. Unfortunately, comparison with UALVP 55261 is not possible as distal tarsal III is not represented in the material of UALVP 55261 that has so far been prepared. However, distal tarsal III is preserved in the indeterminate pachyrhinosaurin TMP 2002.076.0001 and is sub-oval with a proximodistally thickened lateral edge and a rugose anterior edge bearing several concavities.

In proximal view, distal tarsal IV (Fig 6D) appears sub-triangular, with a posteriorly directed apex, a well-defined anteromedial corner, and a beveled anterolateral corner. The lateral edge of the tarsal is sinuous, with the anterior part convex and the posterior part concave. The posteriorly directed apex is more acute than the anteromedial corner. This distal tarsal is very similar in appearance to distal tarsal IV of *Centrosaurus* UALVP 55261. In both cases, distal tarsal IV bears a small concavity on the distal surface of the posterior apex, and a second, although much shallower, concavity near the anterior end of the thick medial edge. The central part of the convex distal surface displays a very shallow depression, although this feature is slightly less pronounced in UALVP 42 than in UALVP 55261.

Though there are striking similarities in the morphology of distal tarsal IV between UALVP 42 and UALVP 55261, this element does show variation among taxa. Distal tarsal IV of *Styracosaurus* TMP 1989.097.0001 is approximately quadrilateral rather than sub-triangular in outline, and has a pronounced concavity on the posterior face. The bone just proximal to the concavity forms a prominent overhanging lip, as in UALVP 42. As in both UALVP 42 and UALVP 55261, there is a slight depression on the otherwise convex distal surface. Furthermore, its anterior edge extends slightly farther laterally than the posterior edge, which is reminiscent of the laterally directed corner in UALVP 42 and UALVP 55261. However, the outline of the *Styracosaurus* distal tarsal IV in proximal view is more rectangular. A distal tarsal IV of *Wendiceratops* (TMP 2011.051.0017) is similar in morphology to those of UALVP 42 and UALVP 55261, although its edges are weathered and broken so that only the general shape is discernable. MOR 456 8-8-87 (*Einiosaurus*) seems to be a distal tarsal IV but has a reduced posterior corner with a lip on the proximal rather than distal side. *Triceratops* ROM 1434 also preserves a distal tarsal IV, closely appressed to the proximal end of metatarsal IV. The distal tarsal is more rounded in overall shape than its counterpart in UALVP 42.

#### Metatarsals.

**Fig 7:**
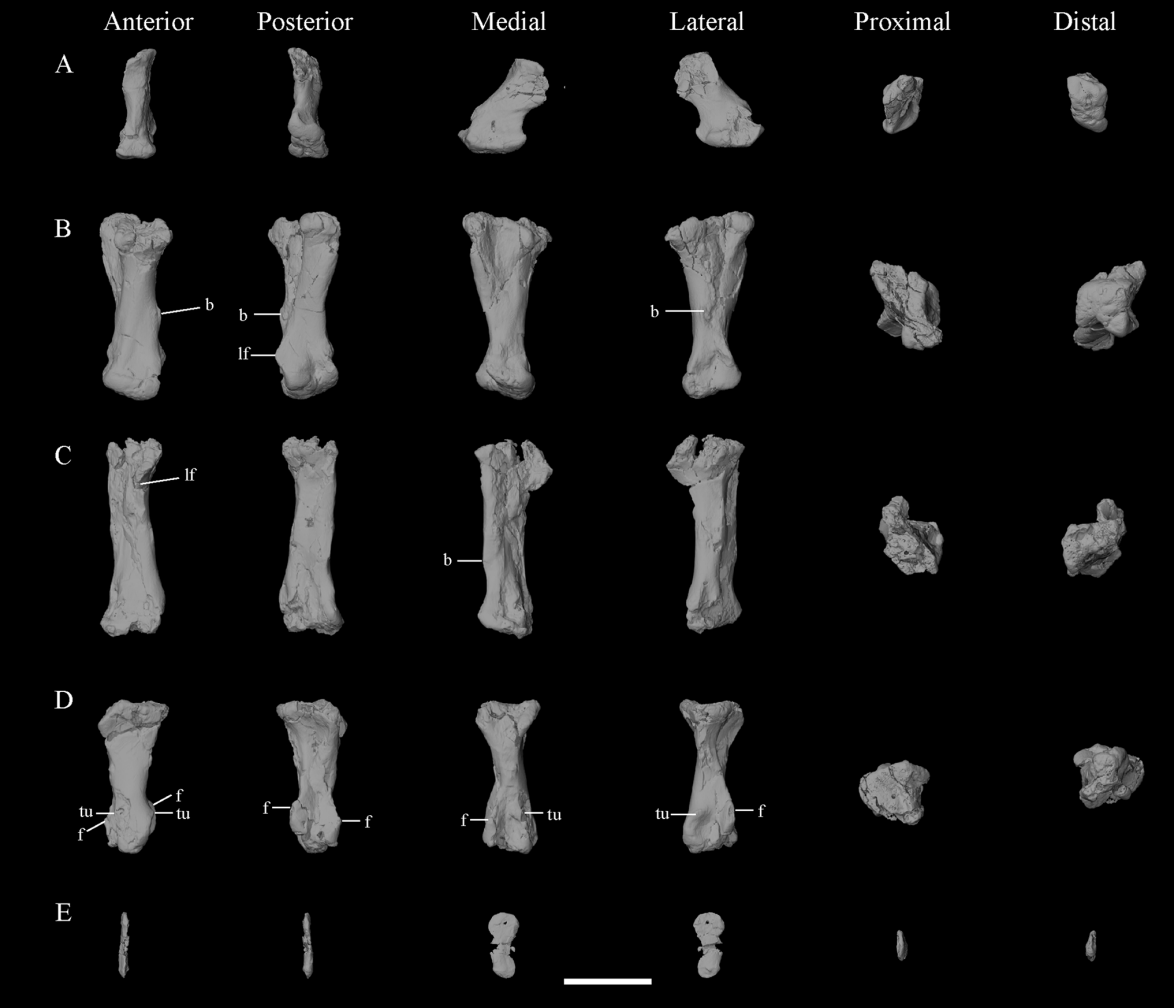
Orthographic views of the metatarsals of the indeterminate ceratopsid UALVP 42. Scale bar = 10 cm. Shown in anterior, posterior, medial, lateral, proximal, and distal views. (A) Metatarsal I. (B) Metatarsal II. (C) Metatarsal III. (D) Metatarsal IV. (E) Metatarsal V. Abbreviations: b = bulge. f = flange. tu = tuberosity.

In UALVP 42, the metatarsals range from 75.6 mm to 215.1 mm in length (Table 3). Metatarsal I (Fig 7A) has a distinctive, asymmetrical shape. The anterior surface of the shaft is longer than the strongly concave posterior surface, which is directed slightly laterally. Because of the discrepancy in length between the anterior and posterior surfaces, the distal end is deflected slightly posteriorly. The proximal end of the metatarsal in UALVP 42 appears to have been crushed mediolaterally and is now slightly thinner in that dimension than the rest of the bone. In undistorted specimens (e.g., *Centrosaurus* AMNH FARB 5351; indeterminate pachyrhinosaurin TMP 2002.076.0001), the proximal surface is flat and about as mediolaterally wide as the shaft of the metatarsal. The distal end is rounded and flares out both posteriorly, creating a hook-like appearance in lateral view, and laterally. The convexity of the distal articular facet wraps onto the anteromedial surface of the bone, giving the distal end a thickened appearance on that side. This thickening is usually most pronounced on the anterior corner of the distal end (e.g., *Chasmosaurus* ROM 839, *Styracosaurus* TMP 1989.097.0001), but that corner has been lost taphonomically in UALVP 42.

**Table 3:** Measurements of metatarsals of UALVP 42.

Element	Length (Proximodistal) (mm)	Width (Mediolateral) (mm)	Depth (Anteroposterior) (mm)
Metatarsal I	124.5	23.4	46.3
Metatarsal II	200.2	42.5	55.1
Metatarsal III	215.1	37.6*	33.0
Metatarsal IV	166.3	45.9	33.6
Metatarsal V	75.6	9.5	23.3

Note: Widths were measured at midshaft. Asterisk indicates that the measurement is slightly unreliable due to oblique crushing.

Metatarsal II (Fig 7B) is almost twice as long as metatarsal I (Table 3), and much more similar to metatarsals III and IV in appearance. The proximal end has an hourglass-like shape, with the anterior and posterior margins expanded relative to the middle of the metatarsal end. The proximal end of metatarsal II has a similar outline in most ceratopsids, but *Avaceratops* and “*Brachyceratops*” reportedly lack a concavity on the lateral side, resulting in a C-shape rather than an hourglass shape [14]. The anterior margin is the widest part of the proximal end, and the anterolateral corner is extended anteriorly, giving the proximal end the appearance of being sheared laterally. Additionally, the entire proximal end is directed slightly anteriorly. The anterior surface of the metatarsal shaft bears a slight, laterally positioned bulge in the midshaft region. The collateral ligament pits on both sides of the distal end are relatively well developed, and a broad flange projects laterally from the proximoposterior corner of the lateral pit. The distal end of the bone angles slightly medially. The distal face of the lateral condyle is somewhat larger anteroposterialy than that of the medial condyle, though the distal end is not ginglymoid.

Metatarsal III (Fig 7C) is the longest of the metatarsals but is not much longer than metatarsal II (Table 3). The medial part of the anterior side of the shaft appears crushed in, and the proximolateral part of the posterior side has been slightly crushed, as has the lateral distal condyle. The proximal end of the metatarsal is widest mediolaterally near the posterior margin and quite narrow at the anterior margin, although this difference is likely exacerbated by, or even the result of, deformation. In most ceratopsid specimens the proximal end of metatarsal III is D-shaped, with the convex side directed laterally (e.g., *Centrosaurus*, ROM 1426; *Triceratops* ROM 1434). In UALVP 42, the D-shape is poorly defined, although the medial margin of the proximal end is slightly concave and the lateral margin is slightly convex. As with metatarsal II, the entire proximal end is angled slightly anteriorly. The distal end has shallow collateral ligament pits, like that of metatarsal II, but lacks the flange seen near the lateral pit on metatarsal II (although there is a slight bulge posteroproximal to the medial pit). Given the deformation to the lateral condyle, any flange that may have originally been present would likely have been crushed.

The proximal end of metatarsal IV (Fig 7D) is concave and has an outline in the shape of a reversed “D”. Although the proximal ends of metatarsals III and IV are somewhat similar in outline, they differ in that the proximal end of metatarsal IV is concave, has a more protrusive convex lateral side, and has a beveled anteromedial corner.

Metatarsal IV is narrowest, both anteroposteriorly and mediolaterally, at the midshaft but expands at the distal end. As with metatarsal III, the proximolateral part of the posterior surface of the shaft has been crushed inward. The collateral ligament pits are larger in metatarsal IV than in any of the other metatarsals, although this could be due to taphonomic distortion. In fact, the distal end is unusual in that the anterior portion is much narrower mediolaterally than the posterior portion. It seems that the distal end of metatarsal IV has been pressed against the distal end of metatarsal III, resulting in the deformation of these areas in both bones, based on how closely they fit together. In other ceratopsids (e.g., *Chasmosaurus* ROM 839, *Pachyrhinosaurus* TMP 1987.055.0186), the anterior and posterior parts of the distal end of metatarsal IV are similar in mediolateral width. Flanges resembling the lateral flange in metatarsal II are situated on the posterior edges of both collateral ligament pits of metatarsal IV, the medial flange being the larger of the two. A slight tuberosity is present at the anteroproximal corner of each pit.

Metatarsal V (Fig 7E) is vestigial, having been reduced to a small, mediolaterally flat splint of bone with the proximal end slightly anteroposteriorly wider than the distal end. In *Chasmosaurus*, the discrepancy in width is more pronounced, judging by ROM 839 and UALVP 52613. Much of the midshaft of metatarsal V in UALVP 42 has been damaged taphonomically, and small projections or fragments of bone protrude from the anterior and posterior edges of the midshaft. In other ceratopsids (*Centrosaurus* AMNH FARB 5351; *Chasmosaurus* ROM 839, UALVP 52613; *Vagaceratops* CMN 41357) the expanded proximal end of the metatarsal causes the anterior edge of the shaft to be concave, a condition which is again especially well developed in *Chasmosaurus*. The posterior edge is straight in *Chasmosaurus* (ROM 839 and UALVP 52613) and very slightly concave in *Vagaceratops* (CMN 41357). Though the taphonomic damage and small projections or fragments of bone on the midshaft make it a little uncertain which side of the metatarsal in UALVP 42 should be the anterior side, one side does appear more strongly concave and this side has therefore been positioned anteriorly. Regardless of whether the correct side is shown anterior here, it should not greatly impact biomechanical studies, as metatarsal V is almost certainly a vestigial structure that would not have played a role in locomotion. The distal end of metatarsal V does not show any indication of an articular surface for contact with a phalanx, as expected given the lack of digit V phalanges throughout ceratopsians. This can help distinguish a metatarsal V from a metacarpal V, as the distal end of a metacarpal V widens to articulate with a manual phalanx.

#### Phalanges.

**Fig 8:**
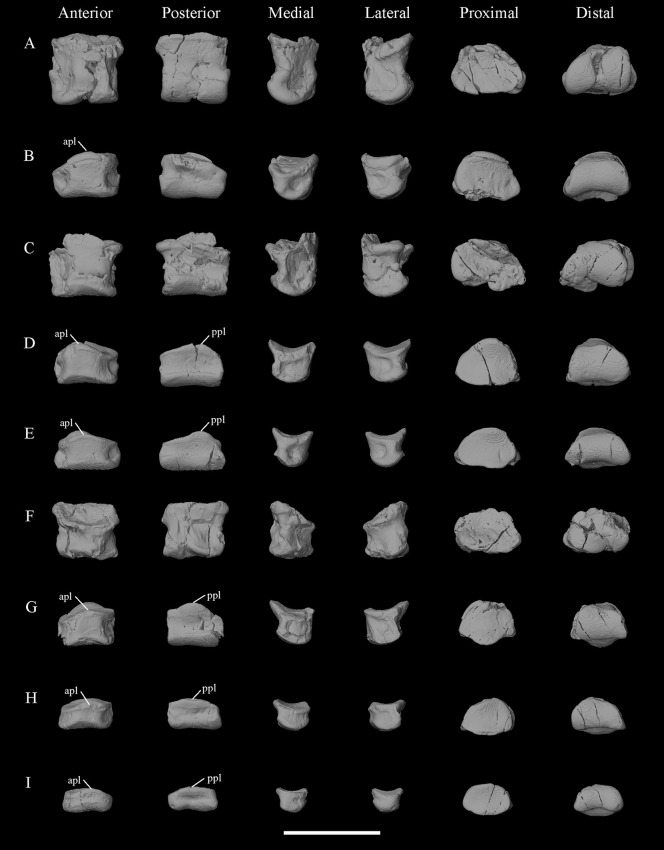
Orthographic views of phalanges of the indeterminate ceratopsid UALVP 42. Scale bar = 10 cm. Shown in anterior, posterior, medial, lateral, proximal, and distal views. (A) Phalanx II-1. (B) Phalanx II-2. (C) Phalanx III-1. (D) Phalanx III-2. (E) Phalanx III-3. (F) Phalanx IV-1. (G) Phalanx IV-2. (H) Phalanx IV-3. (I) Phalanx IV-4. Abbreviations: apl = anterior proximally projecting lip. ppl = posterior proximally projecting lip.

Although phalanx I-1 is missing, the original phalangeal formula of UALVP 42 was presumably 2-3-4-5-0, as in other ceratopsids (e.g., [12,51,52]). The phalanges are shown in Fig 8, and their measurements are given in Table 4. The phalanges of UALVP 42 seem to be divisible into three morphological categories: the proximalmost phalanx in each digit, the other non-ungual phalanges, and the unguals. Progressively distal non-ungual phalanges decrease monotonically in length. Additionally, phalanges in corresponding proximodistal positions are longer in the more medial digits and shorter in the more lateral digits (for example, phalanx III-1 is longer than phalanx IV-1); the only exception is that phalanx IV-2 is slightly longer than phalanx III-2.

**Table 4:** Measurements of non-ungual phalanges of UALVP 42.

Element	Length (Proximodistal) (mm)	Width (Mediolateral) (mm)	Depth (Anteroposterior) (mm)
Phalanx I-1	95.6	64.4	51.4
Phalanx II-1	61.2	70.3	51.3
Phalanx II-2	38.7	71.0	34.8
Phalanx III-1	45.3	68.9	52.8
Phalanx III-2	34.7	67.7	49.8
Phalanx III-3	32.1	69.6	42.4
Phalanx IV-1	40.3	60.0	43.6
Phalanx IV-2	34.8	57.8	46.3
Phalanx IV-3	25.9	55.1	39.3
Phalanx IV-4	21.0	51.7	32.4

Note: Length measured from the middle of the concave proximal end to the middle of the distal end. Mediolateral width measured at the level of the collateral ligament pits, including any projections or flanges when present. Anteroposterior depth measured at the proximal end.

The proximal phalanges have strongly concave proximal ends, whereas the more distal phalanges have proximal ends that are concave anteroposteriorly but convex mediolaterally. The only exception is the ungual of digit IV, which has a proximal end that is concave in all directions, as in the proximal phalanges. The more distal non-ungual phalanges also have convex, proximally projecting, variably prominent lips at the anterior and posterior margins of their proximal ends. None of the phalanges display a clear midline ridge that could be said to define separate cotyles.

**Fig 9:**
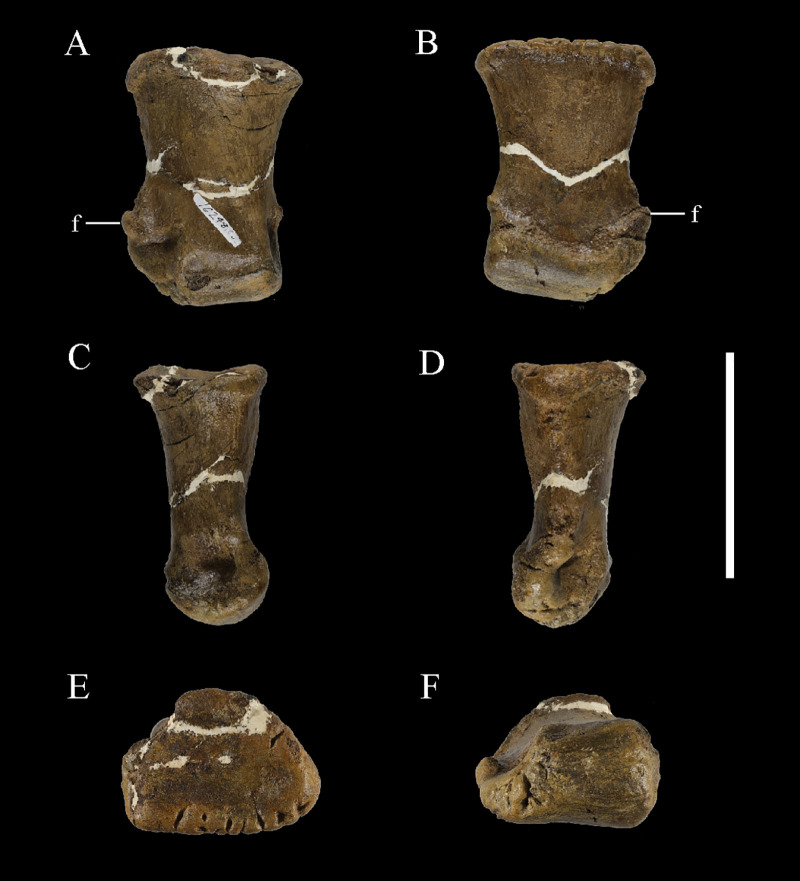
Phalanx I-1 of *Centrosaurus* UALVP 16248. The specimen was photographed using a Canon 80D with the EF 100 mm 1:2.8L IS USM. The effect of telephoto compression from the setup roughly approximates an orthographic projection [16]. Scale bar = 10 cm. Shown in (A) anterior, (B) posterior, (C) medial, (D) lateral, (E) proximal, and (F) distal views. Abbreviations: f = flange.

As noted previously, phalanx I-1 is the only bone missing from the foot of UALVP 42. However, phalanx I-1 generally seems to share a similar morphology across the taxa in which this bone is known (e.g., *Centrosaurus* cast of AMNH FARB 5351 held at the CMN, UALVP 16248; *Chasmosaurus* ROM 839; indeterminate pachyrhinosaurin TMP 2002.076.0001; and *Pachyrhinosaurus* TMP 2004.072.0009), so a phalanx from *Centrosaurus* UALVP 16248 is depicted in our foot reconstruction (Fig 3) and detailed in Fig 9. This element differs from the proximal phalanges of the other digits in being comparatively long (approximately as long as metatarsal I) with an asymmetrical distal end. The asymmetry is caused by a large medially projecting flange posterior to the medial ligament pit that contrasts with a much smaller laterally projecting flange posterior to the lateral pit, although this asymmetry is less pronounced or even absent in some specimens (e.g., *Chasmosaurus* CMN 2245; indeterminate chasmosaurine CMN 8547). As in the proximal phalanges of UALVP 42, the proximal end of phalanx I-1 is concave.

In all non-ungual phalanges of UALVP 42 (Fig 8), the proximal surface is wider mediolaterally than anteroposteriorly. The posterior surface of the shaft of each non-ungual phalanx is concave proximodistally and separated from the rounded distal end by a sharp transverse ridge. Additionally, all non-ungual phalanges have medial and lateral collateral ligament pits. In some of the phalanges, such as phalanx II-1, the ligament pit is ventrally rimmed by a flange. In phalanx III-2, most of the lateral flange is subdued, but the proximalmost part of the flange flares abruptly to create the appearance of a small process projecting out from just posterior to the ligament pit. Non-ungual pedal phalanges in other ceratopsid taxa often have flanges adjacent to the ligament pits, but these features vary widely in presence and form within and between taxa. The exception is that in all taxa the more distal phalanges of digit IV are less likely to show any type of flange (e.g., *Centrosaurus* cast of AMNH FARB 5351 held at the CMN; *Chasmosaurus* ROM 839, UALVP 52613; indeterminate centrosaurine CMN 8896; indeterminate pachyrhinosaurin TMP 1989.09.0001; *Utahceratops* UMNH 20444; *Vagaceratops* CMN 41357).

The distal ends of most of the non-ungual phalanges of UALVP 42 exhibit some asymmetry. In digit II, the distal ends of the phalanges are anteroposteriorly wider on the lateral side, although only slightly so in the case of phalanx II-2. In digit III, this same type of distal end asymmetry is present in phalanges III-1 and III-3. The medial and lateral sides of the distal end of phalanx III-2 appear subequal in anteroposterior width, but the convexity of the anterior edge of the distal end is steeper on the lateral side and gentler on the medial side. As preserved, the distal end of phalanx IV-1 is triangular, but this strange morphology is likely the result of crushing of the anterior part of the distal end. The proximal end of phalanx IV-2 does not show corresponding compression, resulting in slight incongruity in the articulation of the two bones. The distal ends of phalanges IV-2 and IV-3 are asymmetrical, with the medial side anteroposteriorly wider. The medial and lateral sides of the distal end of phalanx IV-4 are subequal in anteroposterior width. Overall, the distal ends of the non-ungual phalanges widen anteroposteriorly on the side closer to an imaginary parasagittal plane between digits III and IV. Similarly, the shafts of phalanges II-1 and III-1 have medial sides that are gently sloping, and lateral sides that are nearly sheer. Although phalanx IV-1 of UALVP 42 is too damaged to assess the steepness of the sides, in *Utahceratops* UMNH 20444 the medial side of the corresponding phalanx is sheer while the lateral side is gently sloped, as the pattern would dictate. The same pattern in the phalanges of *Centrosaurus* was noted by [5], suggesting that it may be common throughout Ceratopsidae.

The proximal ends of the non-ungual phalanges of UALVP 42 also vary morphologically. Many of the specific points of variation are shared with other ceratopsid specimens, such as *Styracosaurus* TMP 1989.097.0001 (based on Appendix D in [11]), *Centrosaurus* UALVP 55261, and *Utahceratops* UMNH 20444, suggesting that they represent consistent differences among individual elements rather than distortion or individual variation. Some of this variation is evident in the anterior and posterior views of the phalanges. In phalanx II-1, the proximolateral corner is beveled. In phalanx II-2, the anterior edge of the proximal surface medial to the proximally projecting lip recedes farther distally than the edge lateral to the lip. Though this part of the anterior edge is only weakly concave, in *Styracosaurus* TMP 1989.097.0001 and *Utahceratops* UMNH 20444, this part of the anterior edge is distinctly concave. In phalanges III-2 and III-3, the anterior and posterior proximally projecting lips are medially located, rather than centrally located as in phalanges IV-2, IV-3, and IV-4. In phalanges IV-2 and IV-3, the anterior edge medial to the proximally projecting lip recedes farther distally than the edge lateral to the lip, as in phalanx II-2. Finally, in phalanx IV-4 the anterior edge medial to the proximally projecting lip is slightly concave, while the lateral part of the edge is slightly convex and recedes farther distally than the medial edge.

Other points of variation are most readily observed in proximal view of the phalanges. In all non-ungual phalanges except IV-3 and IV-4, the posteromedial corner is beveled. In phalanx IV-3, the posterolateral corner is instead beveled, and in phalanx IV-4 both posterior corners are beveled. In phalanx III-2, the entire posterior edge is smoothly convex, whereas in phalanx III-3 the posterior edge is laterally straight with a narrow medial convexity. In phalanx IV-2, the posterior edge is more prominently convex than in phalanx III-2, with a slight indentation on the medial side. Additionally, the posterior proximally projecting lip of phalanx IV-2 projects farther posteriorly than in the other phalanges in which this feature is present (the more distal non-ungual phalanges).

**Fig 10:**
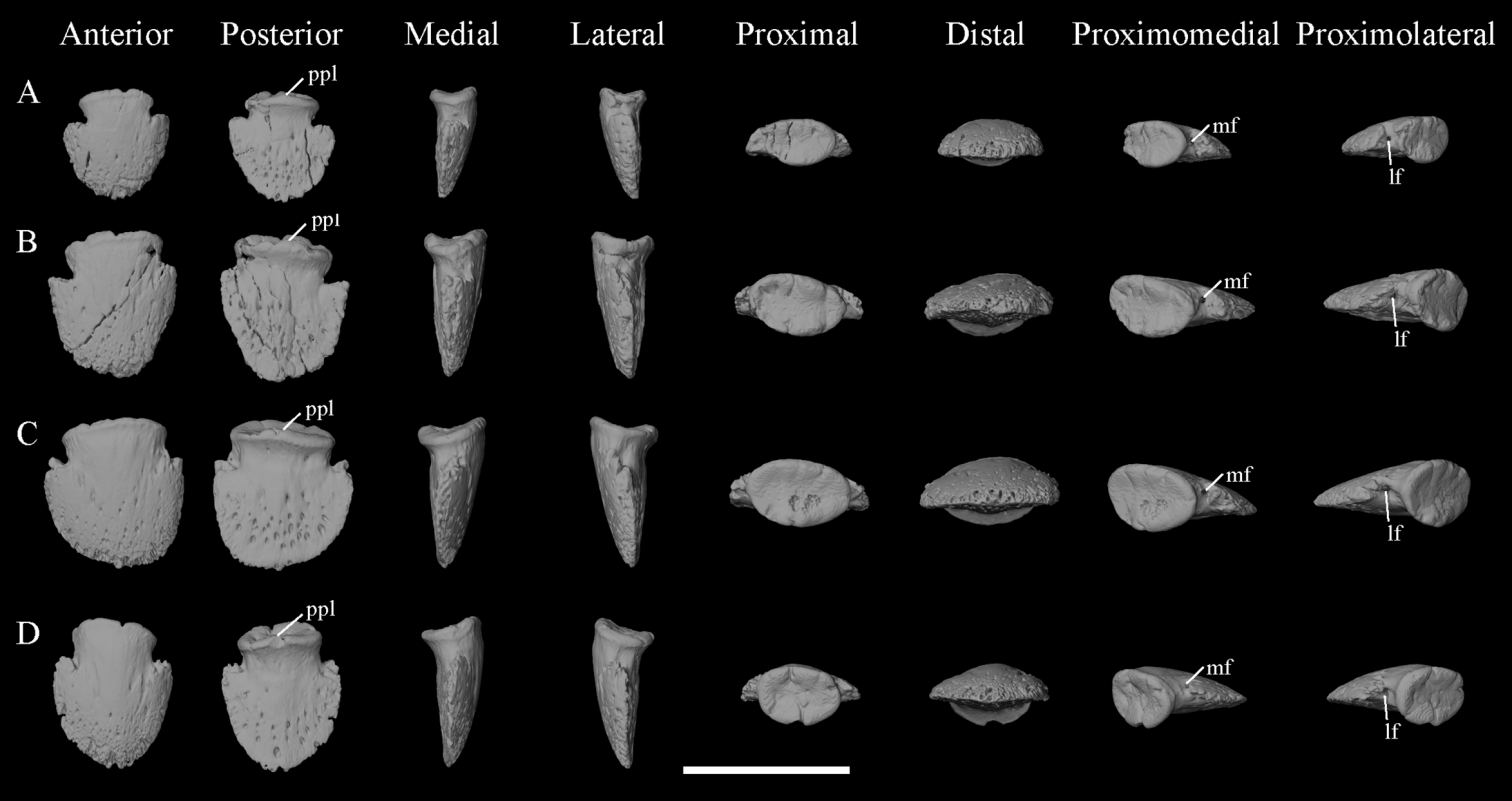
Orthographic views of the unguals of the indeterminate ceratopsid UALVP 42. Scale bar = 10 cm. Shown in anterior, posterior, medial, lateral, proximal, distal, oblique proximomedial, and oblique proximolateral views. (A) Ungual from digit I. (B) Ungual from digit II. (C) Ungual from digit III. (D) Ungual from digit IV. Abbreviations: lf = lateral foramen. mf = medial foramen. ppl = posterior proximally projecting lip.

The unguals of UALVP 42 are shown in Fig 10, and their measurements are given in Table 5. As has been noted previously for ceratopsids (e.g., [1]), the unguals are hoof-like. The middle of the posterior margin of the proximal end has a proximally projecting lip as in some non-ungual phalanges, but this feature is poorly developed, while the anterior margin lacks a distinct projection. Unguals II and IV are slightly longer than wide, whereas unguals I and III are about equally wide and long. The unguals appear somewhat spade-shaped in anterior or posterior view, with a short proximal shaft that flares abruptly both medially and laterally to form an expanded blade bearing numerous grooves for blood vessels that would have nourished the keratin overlying the ungual blade in life. This vascularization is particularly evident on the posterior side. In some ceratopsids (*Centrosaurus* CMN 1426*; Chasmosaurus* CMN 2245, ROM 839; *Styracosaurus* TMP 1989.097.0001; *Triceratops* ROM 1434, ROM 52428 *Vagaceratops* CMN 41357), at least one ungual displays a visible, posteriorly prominent step between the vascularized blade and the shaft of the ungual on the posterior side, but the shelf is not present in any unguals of UALVP 42. Foramina are present on the medial and lateral sides of each ungual where the blade arises from the proximal shaft. The lateral foramen is placed more posteriorly and the medial foramen more anteriorly. In ungual I, each foramen is close to the anteroposterior midline on its side of the ungual, but in successively more lateral unguals the respective posterior and anterior positions of the two foramina gradually become more extreme.

**Table 5:** Measurements of unguals of UALVP 42.

Element	Length (Proximodistal) (mm)	Width (Mediolateral) (mm)	Depth (Anteroposterior) (mm)
Ungual I	63.8	63.8	28.6
Ungual II	83.0	77.4	36.6
Ungual III	83.9	84.1	41.0
Ungual IV	82.0	70.3	36.0

### Articulation

Fig 3 shows a digital model representing the preserved elements of UALVP 42 in articulation without any retrodeformation or restoration of the individual bones, which is also available in the Supplemental Material. Fig 4 shows a reconstruction of the articulated lower hindlimb as it would have appeared in life. The distal part of the shaft of the fibula articulates with the anterior surface of the lateral malleolus of the tibia, as can be observed in articulated ceratopsid specimens (e.g., *Centrosaurus* AMNH FARB 5351, *Chasmosaurus* UALVP 52613, indeterminate chasmosaurine CMN 8547) and has been previously reported (e.g., [6,45,53]). The anteromedial to posterolateral expansion of the distal part of the fibula creates a close fit between the posterior surface of this expansion and the anterior surface of the lateral malleolus of the tibia. However, as preserved, this configuration leaves a gap between the proximal ends of the tibia and fibula that is unlikely to reflect the original condition (see model in the Supplementary Material). Rotating the digital models of the bones to remove this gap while maintaining the excellent articulation at their distal ends causes their distal halves to overlap in space, creating an impossible geometry. Thus, the gap at the proximal end is likely the result of taphonomic deformation of the tibia and/or fibula, including the mediolateral compression of the proximal end of the tibia noted previously. The articulated specimens mentioned above show that two parts of the proximal end of the tibia should articulate with the fibula: the posterior surface of the lateral flange, and the lateral surface of the lateral condyle. This articulation also matches that described by [45].

The inference that the fibula articulates with the tibia by contacting the posterior surface of the lateral flange proximally, and the anterior surface of the lateral malleolus distally, suggests in turn that a slight offset in the exact direction of mediolateral expansion between the proximal and distal ends of the tibia is the natural condition, as seen in *Centrosaurus* UALVP 55261. As configured in the model, the proximal end of the fibula lies posterior to the lateral flange of the tibia with a gap between the fibula and the preserved part of the proximal end of the tibia so that, were the tibia undistorted, the fibula would be in natural contact (compare Figs 3 and 4). It should also be noted that the fibula extends slightly farther proximally than the tibia when both bones are in articulation with the proximal tarsals as described below. This condition is also the one most commonly seen in specimens with an articulated tibia and fibula (e.g., right hindlimb of *Chasmosaurus* ROM 839, indeterminate chasmosaurine CMN 8547, *Styracosaurus* TMP 1989.097.0001), and was described by [6] for *Centrosaurus*, though [45] reported that in several chasmosaurines the tibia and fibula extended equally far proximally.

The astragalus articulates with the medial malleolus of the tibia. As noted previously, the proximally prominent surface on the anterior part of the astragalus rests against a shelf on the anterior edge of the distal face of the medial malleolus. The lateral face of the astragalus abuts the lateral malleolus of the tibia so that the lateral projection of the astragalus partially covers the anterodistal face of the lateral malleolus. As reconstructed by Sternberg, the distal end of the tibia extends medially beyond the astragalus. This condition is generally seen in chasmosaurines (e.g., *Chasmosaurus* CMN 2245, *Triceratops* in [4]), while in centrosaurines the medial edges of the tibia and astragalus are usually in alignment (e.g., *Centrosaurus* in [6], indeterminate pachyrhinosaurin TMP 2002.076.0001), although exceptions occur in both groups (e.g., *Chasmosaurus* ROM 839, an indeterminate centrosaurine CMN 8896).

The calcaneum articulates closely with the distal end of the fibula, and as noted previously, there is evidence of possible fusion between these elements in UALVP 42. The calcaneum is rotated such that its medial surface faces somewhat posteriorly and contacts the anterior surface of the lateral malleolus of the tibia. In fact, the concave medial surface of the calcaneum closely matches the opposing convex surface of the lateral malleolus. Although the astragalus and calcaneum are reportedly fused in two ceratopsians [6,47], they are separate in UALVP 42. In many ceratopsid specimens with the proximal tarsals preserved in articulation with the tibia and fibula, a small gap is visible between the astragalus and calcaneum that may have been filled with cartilage in life (e.g., *Centrosaurus* AMNH FARB 5351; *Chasmosaurus* CMN 2245, UALVP 52613; *Vagaceratops* CMN 41357), so that condition is reflected in our reconstruction.

The natural articulation of the calcaneum with the fibula orients the calcaneum as a vertical disc covering most of the anterior surface of the lateral malleolus of the tibia and only partially covering the tibia’s distal end. Taken together, the astragalus, lateral malleolus of the tibia, and calcaneum create a roller surface that opposes the surface formed by the proximal faces of the distal tarsals and the proximal ends of the metatarsals (Fig 3G-H). In distal view, the crural and proximal tarsal roller surface has a distinctly concave anterior margin [16].

There is some uncertainty regarding the exact position of the distal tarsals on the proximal ends of metatarsals III and IV, but we suggest the following arrangement based on comparisons with other ceratopsid specimens. In ceratopsids in which the distal tarsals are preserved in place (including *Chasmosaurus* UALVP 52613) the larger distal tarsal tends to be laterally positioned [5, text of 6] so that arrangement is adopted here. The larger distal tarsal (distal tarsal IV) is over, and almost entirely covers, the proximal end of metatarsal IV, though the posterior corner protrudes posteriorly, and the smaller distal tarsal (distal tarsal III) covers the posterior half of the proximal end of metatarsal III with the posterior edge of distal tarsal III protruding slightly posteriorly (Fig 3h). Similar placement of the distal tarsals is observable in articulated ceratopsid hindlimb specimens that include distal tarsals (*Centrosaurus* AMNH FARB 5351, *Chasmosaurus* UALVP 52613; *Styracosaurus* TMP 1989.097.0001 is a possible counterexample, but given that there had been some confusion regarding the phalangeal formula as a result of incorrect reassembly after preparation, the distal tarsals in that specimen may be displaced). Likewise, the posterior portions of both distal tarsals overhanging just slightly beyond the proximal surfaces of metatarsals III and IV matches the condition observed in UALVP 52613 (*Chasmosaurus*).

Though each distal tarsal has a concave face and a convex face, which should be proximal and which should be distal is not readily apparent. The proximal surfaces of the distal tarsals are reportedly concave in the non-ceratopsid neoceratopsians *Leptoceratops* [54] and *Protoceratops* [51]. Distal tarsal III is preserved in articulation with metatarsal III in AMNH FARB 5351 (*Centrosaurus*) and TMP 2002.076.0001 (an indeterminate pachyrhinosaurin) and has a convex proximal surface. Distal tarsal IV is preserved in articulation with metatarsal IV in AMNH FARB 5351 (*Centrosaurus*) and ROM 1434 (*Triceratops*) and has a concave proximal surface. Note that distal tarsal III is missing in ROM 1434 (*Triceratops*) and distal tarsal IV is missing in TMP 2002.076.0001 (an indeterminate pachyrhinosaurin). The proximal articular surfaces of the individual distal tarsals in other cerapodan dinosaurs are variously convex, concave, and flat (e.g., *Convolosaurus* in [55], *Diluvicursor* in [56], *Gasparinisaura* in [57], *Goyocephale* in [58], *Haya* in [59], *Iguanodon* in [60], *Jeholosaurus* in [61], *Lesothosaurus* in [34], and *Tenontosaurus* in [39]), and the condition may differ between distal tarsal III and distal tarsal IV of an individual. In our reconstruction of UALVP 42, we have positioned distal tarsal III with the convex face directed proximally and distal tarsal IV with the concave face directed distally to be consistent with the orientations of the distal tarsals in AMNH FARB 5351 (*Centrosaurus*), ROM 1434 (*Triceratops*), and TMP 2002.076.0001 (an indeterminate pachyrhinosaurin). Additionally, the orientation of distal tarsal III, with the long axis of the tarsal being oriented approximately mediolaterally, matches the orientation of this element as preserved in UALVP 52613 (a juvenile *Chasmosaurus*) and TMP 2002.076.0001 (an indeterminate pachyrhinosaurin). In TMP 2002.076.0001 the proximodistally thicker edge of distal tarsal III is positioned laterally, another point reflected in our reconstruction. The orientation adopted here for distal tarsal IV, with the most prominent corner directed posteriorly and the other corners directed laterally and proximally, matches the orientation of distal tarsal IV in ROM 1434 (*Triceratops*).

Unlike in many reconstructions of ceratopsid feet, metatarsals I-IV are closely appressed together throughout their entire length, with metatarsal V lying parallel to metatarsal IV and only contacting the latter proximally and distally due to the curvature of the lateral surface of metatarsal IV. This reflects the condition seen in articulated specimens (e.g., *Centrosaurus* AMNH FARB 5351). It is possible that the close fit between the distal ends of metatarsals III and IV achieved in our reconstruction is exaggerated due to deformation. However, based on the condition observable in articulated ceratopsid specimens, there is no evidence that the metatarsals should instead be splayed, or separated by gaps between their shafts, as seen in many restorations (e.g., [5,13,14]). The mode of metatarsal articulation adopted here matches that described for *Triceratops* by [Hatcher 1907] and, in combination with the placement of the distal tarsals, gives the proximal surface of the tarsometatarsus an outline approximating a triangle with the apex directed anteriorly. Near the distal ends of the metatarsals, the laterally directed flange on metatarsal II and the medially directed flange on metatarsal IV lie posterior to metatarsal III. The distal articular surfaces of metatarsals II-IV are all directed almost straight distally, but that of metatarsal I is deflected to face somewhat posterolaterally.

As noted previously, the phalangeal formula is 2-3-4-5-0. The mediolaterally convex proximal ends of the non-proximal phalanges allow for a good articular fit at the interphalangeal joints. The reconstruction shows the metatarsals in the digitigrade posture they presumably adopted in life, with the metatarsophalangeal joints accordingly dorsiflexed. The general accuracy of the reconstruction of the digits, despite the possible effects of taphonomic distortion, may be demonstrated by positioning the foot above an appropriately scaled image of a ceratopsid footprint (modified from Fig 10F in [33]) as seen in Fig 3. As originally articulated, the digits of UALVP 42 generally matched the underlying footprint, though some minor modifications to the interdigital angles (less than 10 degrees) were needed to achieve the level of alignment shown in Fig 3. Especially given the shortness of the proximal phalanges, it is difficult to determine the exact angle at which these elements would have been held relative to the ground.

## Discussion

### Functional morphology

Though most authors identify the lateral flange on the proximal end of the tibia as the cnemial crest, as in the present study [Hatcher 1907, 8, 10, 12, 62, 63], others apply this term to a poorly defined convexity on the anterior surface [6,13,64]. In non-ceratopsid neoceratopsians, the cnemial crest is typically identified as a ridge projecting anteriorly or anterolaterally [65–68], although [69] described a seemingly equivalent ridge as simply part of the lateral condyle that is separated from the rest of the condyle by a sulcus. Given that in these non-ceratopsid neoceratopsians the cnemial crest is sometimes located towards the lateral side of the anterior surface of the tibia [68], it seems likely that this ridge-like cnemial crest in non-ceratopsid neoceratopsians is indeed homologous to the more laterally positioned flange conventionally called the cnemial crest in ceratopsids. The lateral placement of the crest and the articulation of the fibula between the cnemial crest and the lateral condyle suggest that either the muscle insertions associated with the crest may have also moved laterally, resulting in an oblique line of action, or the muscle insertions may have remained in place and the cnemial crest itself became an articular structure bracing the proximal end of the fibula.

As ankle dorsiflexion and plantarflexion would have occurred primarily between the proximal and distal tarsals in non-avian dinosaurs [15], the configuration of the tarsal bones has implications for ankle joint mechanics in both UALVP 42 and other ceratopsids. However, it must be acknowledged that the configuration suggested above is uncertain in some respects, particularly with regard to how the distal tarsals were positioned and oriented, and essentially represents a tentative interpretation based on limited information from a small number of specimens. Additional evidence regarding the correct distal tarsal configuration in ceratopsids, especially from specimens with the ankle preserved in natural articulation, would be valuable. Nevertheless, the correctness of the suggested configuration is provisionally assumed below.

The proximal articular surface participating in the joint was formed by the lateral malleolus of the tibia as well as by the astragalus and calcaneum, and had a convex topography and a curved, anteriorly concave outline as noted above and by [16]. The subtriangular opposing distal articular surface was formed medially by the flat proximal ends of metatarsals I and II and more laterally by the proximal faces of distal tarsals III and IV. If the orientations suggested above for the distal tarsal elements are accurate, the proximal face of distal tarsal III was convex while that of distal tarsal IV was concave. Interpreted in isolation, the convex portion of the surface could have increased the flexibility of the articulation, while the concave portion could have increased its stability. Without explicit modelling, however, it is difficult to understand how the proximal articular surface would have interacted with the topographically varied distal surface as a whole, or to estimate the ankle’s range of motion. The range of dorsiflexion may have been increased by the anteriorly concave outline of the proximal articular surface, which would have accommodated the anterior apex of the triangular distal articular surface during this motion. Nevertheless, the anterior point would ultimately have served as a bony stop limiting dorsiflexion, which may have been further restricted by soft tissue structures. No such bony stop would have limited the range of plantarflexion.

The simple concave proximal ends of the proximalmost phalanges of the digits may have allowed considerable freedom of movement at the metatarso-phalangeal joints, though the distal ends of the metatarsals are only slightly convex, with the exception of metatarsal I which has a strongly convex distal end that may have permitted an even greater range of motion. The interphalangeal joints may have been restricted in their mobility to dorsiflexion and plantarflexion, given that the proximal ends of the more distal non-ungual phalanges are concave anteroposteriorly but convex mediolaterally, matching the distal ends of the non-ungual phalanges which, though not ginglymoid, are weakly concave mediolaterally in the middle of the distal end.

### Taxonomic variation

Exploring the morphology of UALVP 42 revealed some potential points of taxonomic variation within Ceratopsidae, including the medial edge of the medial malleolus of the tibia extending past the medial edge of the astragalus in many chasmosaurines (e.g., *Chasmosaurus* CMN 2245) but not in many centrosaurines (e.g., *Pachyrhinosaurus* UALVP 57285), a concavity occupying most of the distal end of the fibula in chasmosaurines (e.g., *Triceratops* ROM 1434) but only the anterior half of the distal end of the fibula in centrosaurines (e.g., *Centrosaurus* ROM 1426), details of the shape of distal tarsal IV as described earlier, and unguals having a shelf between the vascularized blade and the shaft in some ceratopsids (e.g., *Triceratops* ROM 1434). However, additional investigation is needed regarding each of these, which is beyond the scope of the present study. Ontogenetic variation in morphology is not readily apparent, with the possible exception mentioned above regarding the absence of an anterior notch in the calcaneum of the juvenile “*Brachyceratops*” specimen USNM V 7957. But the additional investigation needed to verify what, if any, ontogenetic trends are present in the ceratopsid hindlimb is also beyond the scope of this paper.

Given the taxonomic variation in the extent of the concavity on the distal end of the fibula, which tends to be much larger in chasmosaurines, the large size of the concavity in UALVP 42 may suggest that this specimen is referable to Chasmosaurinae. This identification is arguably supported by the presence of a *Chasmosaurus* skull (UALVP 40) near the site where UALVP 42 was collected [70, and Sternberg’s 1920 field notes]. Given that many ceratopsid bonebeds only preserve one ceratopsid taxon, the close association of these two specimens may support their conspecificity. However, few chasmosaurine fibulae have so far been documented, and the proximity between UALVP 42 and UALVP 40 is no more than circumstantial evidence, so referral of the former specimen to Chasmosaurinae is still tentative at best.

## Conclusions

Study of UALVP 42, interpreted in light of other ceratopsid material, including articulated skeletons, has clarified some aspects of the structure and articular configuration of the ceratopsid crus and pes. In life, the fibula articulated proximally between a laterally directed cnemial crest and the lateral condyle and crossed in front of the tibia to distally articulate with the anterior surface of the latter bone. Distal tarsals III and IV, the only distal tarsals present, articulated with the proximal ends of the corresponding metatarsals, and likely had proximal faces that were respectively convex and concave. When articulated, the proximal surface of the articulated tarsometatarsus was subtriangular with an anterior apex that appears to have been accommodated during dorsiflexion by the concave anterior margin of the surface formed by the articulated tibia and proximal tarsals. Phalanges demonstrate asymmetry based on the digit to which they belong. The study of UALVP 42 also highlights potential taxonomic variation in the lower hindlimb elements of ceratopsids, such as whether the unguals have a visible shelf between the shaft and blade on their ventral side or not, as in UALVP 42, which may help resolve phylogenetic relationships between ceratopsid taxa. Increased understanding of the anatomy of the distal part of the ceratopsid hindlimb will also inform future biomechanical studies, resulting in a better understanding of ceratopsid locomotion, and provide a starting point for comparisons between the crural and autopodial elements of various ceratopsid taxa.

## Supporting information

S1 File Digital model of the articulated elements of UALVP 42 (lower hindlimb from an indeterminate ceratopsid), without restoration or retrodeformation of the individual bones. Note that the model has been decimated from the one used to create the figures to facilitate easier access and to comply with file size limitations.
(ZIP)
